# Solvent-free dehydration, cyclization, and hydrogenation of linalool with a dual heterogeneous catalyst system to generate a high-performance sustainable aviation fuel

**DOI:** 10.1038/s42004-022-00725-0

**Published:** 2022-09-27

**Authors:** C. Luke Keller, Karan R. Doppalapudi, Josanne-Dee Woodroffe, Benjamin G. Harvey

**Affiliations:** Research Department, Chemistry Division, US NAVY, NAWCWD, China Lake, CA 93555 USA

**Keywords:** Heterogeneous catalysis, Biofuels, Renewable energy, Energy

## Abstract

The development of efficient catalytic methods for the synthesis of bio-based, full-performance jet fuels is critical for limiting the impacts of climate change while enabling a thriving modern society. To help address this need, here, linalool, a terpene alcohol that can be produced via fermentation of biomass sugars, was dehydrated, cyclized, and hydrogenated in a one-pot reaction under moderate reaction conditions. This sequence produced a biosynthetic fuel mixture primarily composed of 1-methyl-4-isopropylcyclohexane (*p*-menthane) and 2,6-dimethyloctane (DMO). The reaction was promoted by a catalyst composed of commercial Amberlyst-15, H^+^ form, and 10% Pd/C. Two other terpenoid substrates (1,8-cineole and 1,4-cineole) were subjected to the same conditions and excellent conversion to high purity *p*-menthane was observed. The fuel mixture derived from linalool exhibits a 1.7% higher gravimetric heat of combustion and 66% lower kinematic viscosity at −20 °C compared to the limits for conventional jet fuel. These properties suggest that isomerized hydrogenated linalool (IHL) can be blended with conventional jet fuel or synthetic paraffinic kerosenes to deliver high-performance sustainable aviation fuels for commercial and military applications.

## Abstract (web summary)

Sustainable aviation fuels offer the opportunity to both reduce CO_2_ emissions and produce fuels with superior properties to conventional petroleum-based fuels. Here, the bio-based monoterpene linalool is converted into an aviation fuel mixture primarily composed of *p*-menthane and 2,6-dimethyloctane at moderate temperatures and pressures using a combination of Amberlyst-15 and Pd/C catalysts. The resulting fuel is demonstrated to have a higher gravimetric heat of combustion, and a lower viscosity compared to petroleum derived Jet-A.

## Introduction

Worldwide annual CO_2_ emissions attributable to aviation are roughly one billion tons or 2% of the global total^1^. Although a minor contributor to CO_2_ emissions compared to ground transportation, the aviation industry is much more challenging to decarbonize due to the extensive power requirements of commercial and military aircraft^2^. Whereas a rapid expansion of ground vehicles powered by electricity^3^, fuel cells^4^, and hybrid technologies is poised to make a large dent in emissions, these technologies are not easily translated to the aviation sector. The industry has responded to this challenge by embracing new methods for the production of sustainable aviation fuels (SAFs) that can be used as drop-in replacements for conventional petroleum-based fuels, while greatly reducing net greenhouse gas emissions^5^. In addition to the environmental necessity of this approach, there is a growing realization that SAFs can be designed to have properties that exceed those of petroleum-based fuels^2^. For example, SAFs typically have a higher gravimetric energy density, better combustion properties, and better low-temperature properties^6^.

Despite the promise of SAF, renewable jet fuels have not yet been broadly implemented due to high initial costs and limited supplies. One route to SAF production that has been widely studied is the fermentation of biomass carbon sources to produce platform chemicals that can then be converted into diverse fuel components^7–9^. Recent DOE estimates suggest that more than a billion tons of waste biomass are available in the US, on an annual basis, for conversion to fuels and chemicals^10^. These feedstocks could allow for the complete replacement of petroleum-based jet fuel with sustainable alternatives. Currently, fermentation of biomass sources coupled with chemical conversion to synthetic fuels cannot compete, on a cost basis, with traditional methods of fuel production from petroleum^11,12^. Furthermore, there are a number of fuel performance factors that need to be considered. For example, full-performance jet fuels must exhibit a high density (>0.775 g mL^−1^), good low-temperature viscosity (<8.0mm^2^ s^−1^) and a high gravimetric net heat of combustion (NHOC > 42.8 MJ kg^−1^)^13^. Although conventional SAFs based on acyclic alkanes have outstanding gravimetric NHOCs, their low densities, and relatively high viscosities, particularly at −40 °C^14^, greatly reduce their viability as drop-in replacements. To address this challenge, our group has focused on the synthesis of SAFs based on cyclic hydrocarbons^15–19^, Terpenes are a particularly promising source of cyclic hydrocarbons that can be generated biosynthetically and have been the subject of intense study over the last several years^7,14,20^.

Much of the work on terpene-based fuels has been centered on methods for the conversion of terpene substrates into fuel blendstocks. For example, Canoria and coworkers developed a method for converting turpentine obtained during paper production into a diesel fuel additive by partially hydrogenating the pinene component^21^. The resulting hydrogenated turpentine mixture showed great promise as a jet fuel additive. Terpenes have also been studied as precursors to high-density fuels for rocket and missile propulsion. This was accomplished through a variety of techniques including dimerization^22,23^, chemoselective hydrogenation of ring-strained terpenes^24^, and cyclopropanation^19^. Recent work by Harvey et al. demonstrated that pinene, limonene, and sabinene, once hydrogenated, could be blended with synthetic paraffinic kerosenes to generate jet fuel that exceeded Jet-A standards^14^. A promising terpene-derived fuel component identified during this research was the hydrogenation product of limonene, 1-isopropyl-4-methylcyclohexane (*p*-menthane).

*p*-Menthane has a high heat of combustion and low viscosity, making it an outstanding SAF target^14,25–27^. The synthesis of *p*-menthane from bio-based sources typically utilizes cyclic terpenes as the starting material. For example, limonene or α-terpinene can be hydrogenated to *p*-menthane in good yields utilizing group ten metals, including nickel^28^. Recent work has led to the development of a variety of catalysts for this transformation, including MOFs, rhenium oxo complexes, and ruthenium nanoparticles^29–32^.

Substrates for the synthesis of *p*-menthane are not limited to cyclic hydrocarbons. Cyclic ethers, alcohols, and acetates can also be efficiently converted into *p*-menthane. For example, geraniol^33–35^, menthol^33,36^, α-terpineol^33^, and nerol^35^, have all been used as precursors to *p*-menthane. Furthermore, ethers, including 1,8-cineole (eucalyptol), can be converted to *p*-menthane by tandem deoxygenation/hydrogenation methods^37,38^. However, most of these methods require either high hydrogen pressure and harsh conditions, such as strong Lewis acids, or expensive catalytic systems based on discrete noble metal complexes. Another platform terpenoid that shows significant promise as a precursor to both *p*-menthane and more complex fuel mixtures is linalool^39,40^.

Linalool is a terpene alcohol commonly found in a number of plants, including lavender and oranges^39,40^. It is also a major component of several essential oils, including coriander, rosewood, and sweet orange^39,40^. Linalool was originally produced from rosewood oil^39^. However, it has grown in industrial relevance, due to its use in the syntheses of vitamins A and E^39^, as well as its applications in the fragrance^39–41^ and agrochemical industries^39,40^. As a result of this increase in demand, synthetic methods for its production have been developed^39^. Today, linalool is commonly synthesized from α-pinene^39,42^. While linalool production has grown in sophistication from its humble origins, synthetic biology researchers are looking to unseat current production methods for this valuable terpene. Recent work has demonstrated the biosynthetic production of linalool from glucose^43^ and glycerol^44^ with metabolically engineered *E. coli*. Linalool has also been successfully produced in yeast^45^. Until recently, yields of linalool from biosynthetic protocols only approached the gram per liter range^46,47^. However, recent work by Usuda and coworkers demonstrated that linalool can be produced *via* fermentation in yields up to 10.9 g/L^48^. While this process has yet to be industrialized, biosynthetic linalool represents a promising platform chemical for SAF production.

**Fig. 1:**
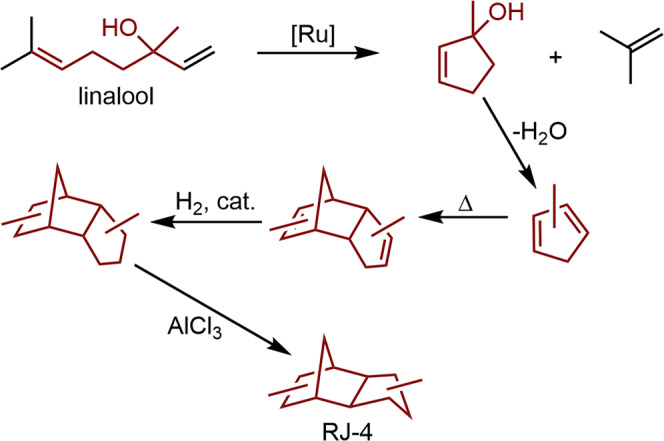
Synthesis of RJ-4 (hydrogenated methylcyclopentadiene dimers) from linalool as described in reference 9. [Ru] is a ruthenium-based metathesis catalyst and the red atoms/structures are the source of bio-based RJ-4.

As an emerging biosynthetic commodity chemical, linalool has been explored as a starting material for a number of other products, including fuels. For example, a study in 2011 demonstrated the conversion of linalool to the high-density missile/jet fuel, RJ-4, which is composed of a mixture of hydrogenated dimethyldicyclopentadiene dimers^9^. Linalool was first converted to 1-methylcyclopent-2-en-1-ol and isobutene through a ring-closing metathesis reaction. Dehydration of the alcohol yielded methylcyclopentadiene, which then rapidly dimerized through a Diels-Alder cycloaddition. Hydrogenation and isomerization of the dimer isomers yielded RJ-4 (Fig. 1). More recent work has shown that RJ-4 can be generated from cellulose by a series of high-throughput chemical steps^49–51^.

Although RJ-4 is an outstanding jet fuel blendstock, it has a relatively high viscosity at −40 °C and cannot be used as a standalone jet fuel^14^. To produce a more diverse jet fuel mixture with lower viscosity and a higher gravimetric heat of combustion, it was of interest to explore the direct dehydration/cyclization of linalool. Some precedence for this approach has been described in the literature. For example, Linares-Palomino et al. described the dehydration of nerol, a linalool isomer, using p-toluenesulfonic acid^52^ while Yu et al.^53^ studied the dehydration of geraniol, linalool, and nerol with Y-zeolites. More recently, Tiefenbacher demonstrated the cyclization of linalool in a supramolecular resorcinarene capsule^35^. This latter approach was particularly interesting because it utilized a hydrophobic pocket to facilitate the cyclization reaction. One drawback of using the resorcinarene capsule was the production of significant quantities of eucalyptol (1,8-cineole) and the somewhat exotic nature of the catalyst. Building on this approach, it seemed reasonable that a macroreticular cation exchange resin would allow us to combine the hydrophobic pocket approach of Tiefenbacher while introducing stronger acid sites that would facilitate the deoxygenation of intermediate oxygenated products like eucalyptol^35,52,53^.

Herein we report two high-throughput, solvent-free procedures for the conversion of linalool to a mixture of *p*-menthane and 2,6-dimethyloctane using commercial catalysts. This approach allows for simultaneous dehydration/cyclization and hydrogenation of the product, resulting in a saturated jet fuel blendstock, which exhibits outstanding gravimetric heat of combustion and low-temperature fluidity.

## Results and discussion

### Two-pot dehydration/hydrogenation of linalool

**Fig. 2:**
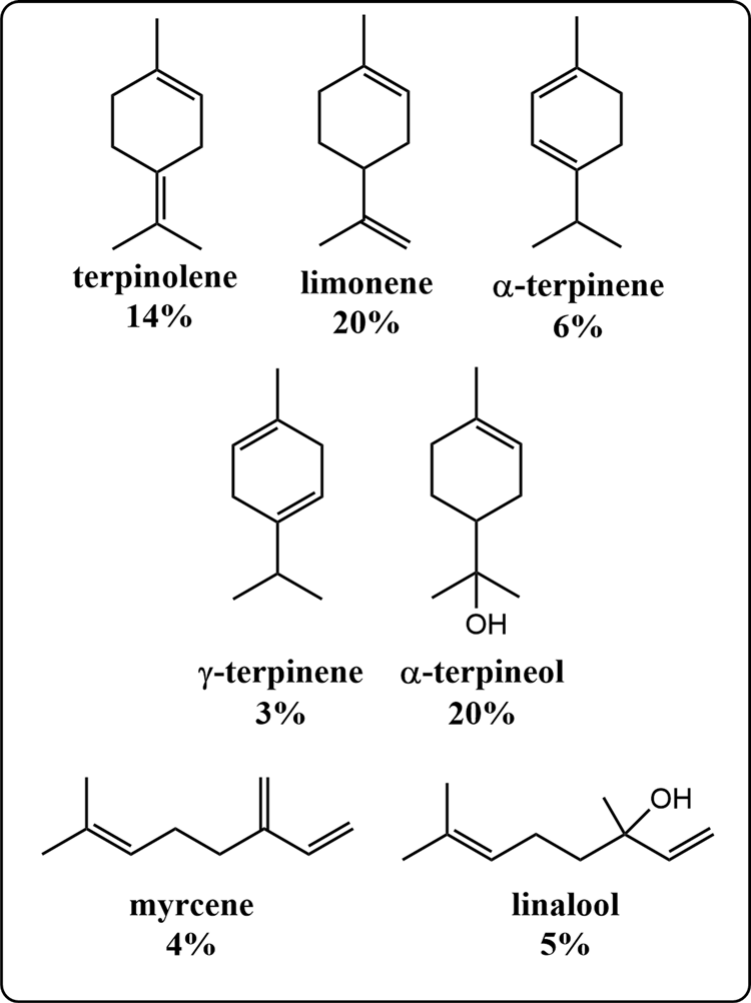
Major products generated by the dehydration of linalool with Amberlyst-15. The composition was determined by GC-FID and accounts for 72% of the product distribution. The other components had similar elution times but were not identified.

**Fig. 3:**
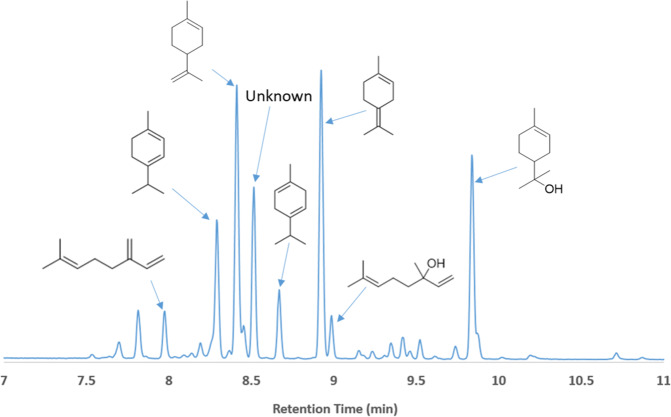
GC chromatogram of the product obtained from dehydration of linalool with Amberlyst-15. Compounds were identified by comparison to the elution times and mass spectra of commercial standards. Traces of dimeric compounds were also observed and can be seen in Figure S1.

To evaluate Amberlyst-15 as a suitable catalyst for the controlled dehydration of linalool, we first studied the reaction at room temperature without any solvent present. A low temperature was desired to eliminate the formation of *p*-cymene through a dehydration/dehydrogenation mechanism^14^. After 16 h, a significant amount of dehydrated, cyclic terpenes was observed by gas chromatography, but the relatively long reaction time and uncontrolled exotherm also resulted in the generation of oligomers not suitable as jet fuel blendstocks. Optimization of the reaction conditions resulted in a straightforward procedure in which the mixture was maintained at 50 °C for 1 h in an oil bath with rapid stirring. The optimized conditions yielded a product mixture consisting of ca. 60% alkenes and 40% oxygenates, with ~8% dimers (Figs. 2, 3, S1, and S2). Alkenes present included myrcene (4%), α-terpinene (6%), limonene (dipentene) (20%), γ-terpinene (3%), and terpinolene (14%). The oxygenated fraction consisted of unreacted linalool (5%), α-terpineol (20%), an unknown compound (11%) with a retention time of 8.52 min (Fig. 2), and small quantities of 1,4-cineole and 1,8-cineole. The mass spectrum of the unknown compound exhibited a peak at 154 *m/z* (Figure S3), confirming that the molecule was an oxygenate, but the retention time of the unknown compound was shorter than the other alcohol products. Furthermore, most terpene alcohols, such as linalool and α-terpineol, show a peak at 136 *m/z*, representing the loss of water. In contrast, the unknown compound did not exhibit a peak at 136 *m/z*, suggesting the lack of an –OH functional group, but instead had a peak at 139 *m/z*, similar to eucalyptol, geranic oxide, and other terpene ethers. Based on the data, we believe the unknown compound is a terpene ether. Additionally, there are a number of small peaks in the GC trace that could not be positively identified, which along with the terpene ether eluting at 8.5 min, comprise ~28% of the total sample. Only a trace of *p*-cymene formed in the reaction, which ultimately allowed for the production of a final fuel product with a high gravimetric heat of combustion.

**Fig. 4:**
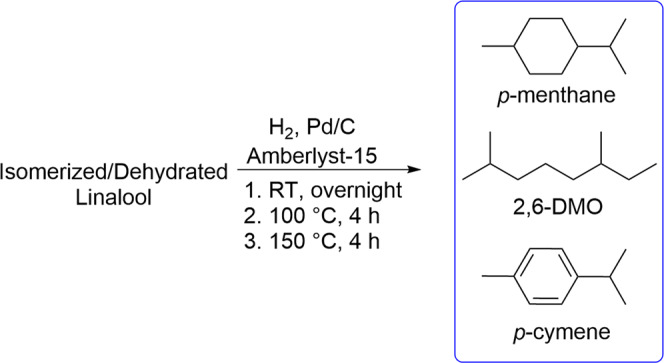
Hydrogenation of isomerized dehydrated linalool. The hydrogenation of isomerized, dehydrated linalool was conducted in a stainless steel autoclave at 500 psi H_2_.

**Fig. 5:**
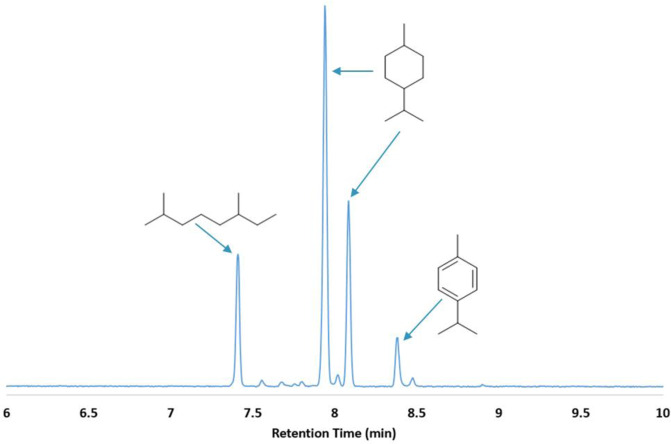
Gas chromatogram (GC-MS) of the final isomerized hydrogenated linalool (IHL) fuel mixture derived from the two-pot method. The mixture is composed of 2,6-DMO, *p*-menthane (two diastereomers), and *p*-cymene.

Although the initial results were promising, it was of interest to develop a tandem dehydration-hydrogenation protocol, through which all of the low molecular weight intermediates could be converted to saturated jet fuel-range products. A dual catalyst system was generated by simply combining Amberlyst-15 with commercial 10% Pd/C. This catalyst system was added to the reaction mixture described above, which was then sealed in a reactor and pressurized with hydrogen. To prevent the formation of *p*-cymene, the hydrogenation reaction was first conducted at room temperature. When the uptake of hydrogen slowed, the temperature was increased first to 100 °C and finally 150 °C to complete the dehydration of α-terpineol, residual linalool, and other oxygenates, while simultaneously hydrogenating the dehydration products (Fig. 4). The reaction mixture was then worked up and distilled to yield a fuel mixture [isomerized hydrogenated linalool (IHL)] composed of 60% *p*- menthane (two diasteromers), 35% 2,6-dimethyloctane, and 4% *p*-cymene *(*Fig. 5*)*. The hydrogenated product distribution suggests that many of the unidentified compounds observed in the chromatogram of the dehydrated product are converted to 2,6-DMO, along with myrcene and residual linalool. The *p*-cymene present in the final product is generated by dehydrogenation of the cyclic terpenes^54,55^. In our hands, the amount of *p*-cymene produced varied widely depending on subtle differences in temperature, heating rates, and hydrogen pressure. In general, the amount of *p*-cymene was minimized by limiting the temperature, allowing for complete hydrogenation of the product mixture before further heating was employed, and keeping the hydrogen pressure constant in the reactor. Alternatively, if desired, the reaction can be tuned to produce higher quantities of *p*-cymene by increasing the temperature or reducing the hydrogen pressure. For example, conducting the hydrogenation at 150 °C without initial steps at lower temperatures resulted in the production of up to 40% *p*-cymene. Aromatic compounds like *p*-cymene are efficient at swelling nitrile rubber elastomers^56^ and can be vital for maintaining engine integrity as well as preventing fires in flight due to fuel leakage. The ability to control the amount of aromatics in the final fuel is a compelling component of this work.

### One-pot dehydration/hydrogenation of linalool

To increase the efficiency of the process, we studied a one-pot reaction for the conversion of linalool to a mixture of *p*-menthane and 2,6-DMO. The same mixed catalyst was used, but a two-stage reaction was conducted to first dehydrate and cyclize linalool and then hydrogenate and further deoxygenate intermediate products. This single pot method resulted in the clean conversion of linalool to *p*-menthane (55%), *p*-cymene (3%), and 2,6-dimethyloctane (41%), similar to the results obtained with the two-pot method.

### Dehydration/hydrogenation of terpene ethers (1,8-cineole; 1,4-cineole)

**Fig. 6:**
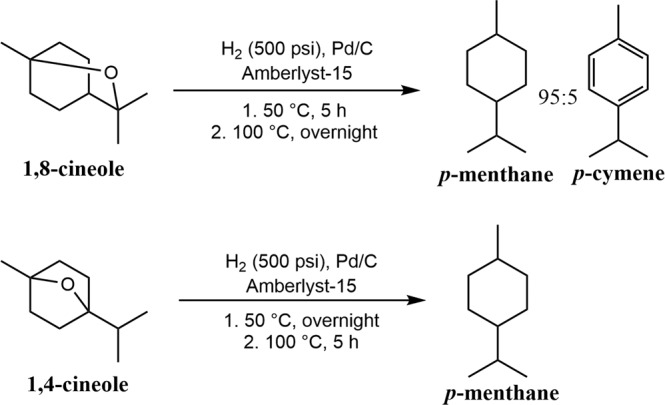
Deoxygenation of 1,8-cineole and 1,4-cineole. Conversions of cyclic ethers to *p*-menthane were conducted in a stainless steel autoclave.

In order to better understand the possible mechanisms and intermediates of the dehydration reaction, and to expand the substrate scope of this method, we attempted the dehydration of 1,8-cineole and 1,4-cineole under similar conditions to the one-pot reaction described above. Amberlyst-15 and 10% Pd/C were added to 1,8-cineole in a stainless steel reactor, which was pressurized to 500 psi of hydrogen and then heated to 50 °C for five h. Subsequently, the reaction temperature was increased to 100 °C for 18 h. The reaction yielded *p*-menthane as the major product, with 5% *p*-cymene present (Fig. 6). 1,4-cineole was dehydrated/hydrogenated under similar conditions. By staging the reaction, first at 50 °C overnight, followed by a slow ramp to 100 °C and five h at that temperature, *p*-menthane was obtained as the sole product (Fig. 6). To the best of our knowledge, this process represents the first synthesis of *p*-menthane directly from 1,4-cineole. Based off these results, we concluded that ethers formed during the linalool dehydration process can easily be further deoxygenated under the reaction conditions and converted to our desired products.

The use of a cheap, commercially available dehydration catalyst and no solvent, represents a significant improvement over previous methods described in the literature. For example, Luska, et al. developed a procedure for the deoxygenation/hydrogenation of 1,8-cineole to *p*-menthane, but their method required high pressures (120 bar), a large quantity of heptane solvent, and a ruthenium nanoparticle catalyst to achieve the same results^38^. Alternately, Song et al. were able to deoxygenate 1,8-cineole at lower temperatures, but their procedure required chlorinated solvents and a palladium-impregnated MOF catalyst^37^. Lin et al. were able to develop a biphasic system for tandem dehydration/hydrogenation of 1,8-cineole to *p*-menthane in high yields, but this method required trifluoroacetic acid as a solvent and low 1,8-cineole concentrations^27^. Finally, Marks et al. developed a procedure for the conversion of 1,8-cineole to *p*-menthane, but their method required the use of exotic Lewis acids to effect deoxygenation^57^. In contrast to existing methods, our approach for deoxygenation/hydrogenation of linalool and cineoles is cost-effective and simple, which may allow for fuel production at commercial scale.

### Fuel properties of IHL

**Fig. 7:**
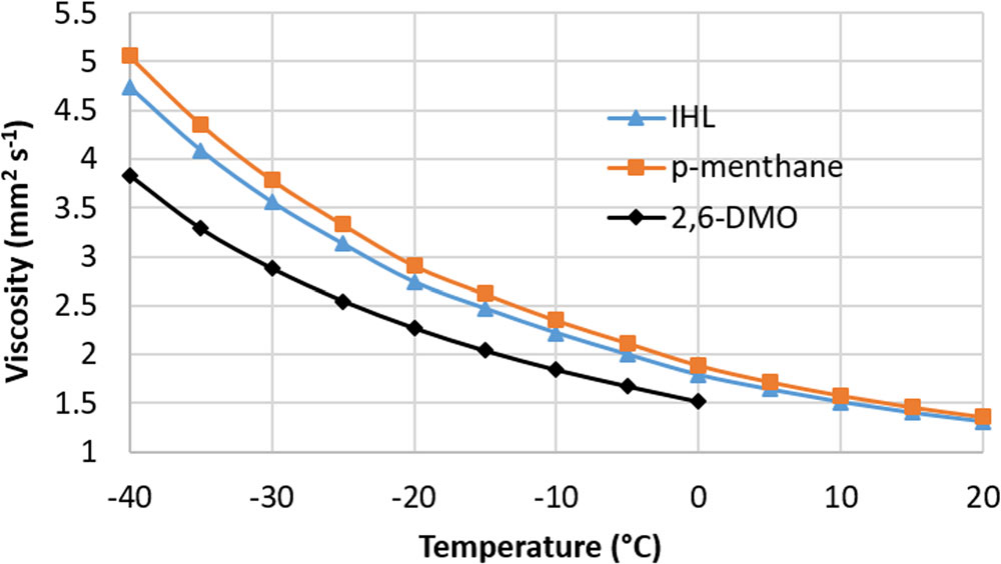
Kinematic viscosity of isomerized hydrogenated linalool (IHL), *p*-menthane and 2,6-DMO. The data for *p*-menthane and 2,6-DMO were taken from a literature source^14^ and are provided to show that IHL, which is a mixture of *p*-menthane and 2,6-DMO, has a viscosity intermediate between the two components across the entire temperature range studied.

After isolating the hydrogenated product mixture obtained from linalool, several key fuel properties were measured and compared to conventional Jet-A (Table 1). The heat of combustion for IHL was 43.54 MJ kg^−1^, which exceeds the Jet-A standard of 42.80 MJ kg^−1^ by 1.7%. The density of the fuel at 15 °C was found to be 0.783 g mL^−1^, which meets the Jet-A requirement. The kinematic viscosity of IHL at −20 °C was found to be 2.74 mm^2^ s^−1^, well below the established maximum for Jet-A. Further, the kinematic viscosity at −40 °C was only 4.74 mm^2^ s^−1^, >60% lower than the maximum allowed viscosity. The properties of IHL were also compared to those of its primary components (*p*-menthane and 2,6-DMO) as well as RJ-4 (Table 1). As expected, the density, heat of combustion, and viscosity of IHL (Fig. 7) are intermediate between those of *p*-menthane and 2,6-DMO. The dehydration/isomerization/hydrogenation procedure generates a mixture that leverages the high density of *p*-menthane, high gravimetric NHOC of 2,6-DMO, and exceptional low-temperature viscosity of both components. Although IHL cannot match the high volumetric NHOC of RJ-4, IHL could be readily blended with linalool-derived RJ-4 to generate a fuel with enhanced volumetric heat of combustion while still maintaining excellent low-temperature properties. Further, IHL can be blended in high concentrations with either conventional jet fuel or other synthetic paraffinic kerosenes to generate high-performance sustainable aviation fuels.

**Table 1:** Fuel properties of isomerized hydrogenated linalool (IHL) and its components compared to RJ-4, HEFA-Jet, and Jet-A.

Fuel	NHOC (MJ kg^−1^)	NHOC (MJ L^−1^)	ρ (15 °C, g mL^−1^)	η (−20 °C, mm^2^ s^−1^)	η (−20 °C, mm^2^ s^−1^)	H content (%)
IHL^a^	43.54	34.10	0.783	2.74	4.74	14.8
p-menthane^b^	43.20	34.72	0.804	2.98	5.19	14.4
2,6-DMO^b^	43.98	32.26	0.733	2.27	3.83	15.4
RJ-4^b^	42.21	39.03	0.925	18.31	49.86	12.4
HEFA-Jet^b^	43.73	33.32	0.762	5.65	12.77	
Jet-A	>42.80	>33.17	>0.775	<8.0	<12.0	>13.5

^a^Fuel properties were evaluated on IHL composed of 60% *p*-menthane, 35% 2,6-DMO, and 5% *p*-cymene. ^b^Fuel properties were taken from reference ^14^.

## Conclusions

Herein, we report a method for the high-throughput conversion of linalool to a viable biosynthetic jet fuel blendstock. The selective procedure can be conducted in a single dual-stage reaction without the addition of solvent, utilizes readily available commercial catalysts, and results in good yields of a saturated fuel product containing primarily *p*-menthane and 2,6-dimethyloctane, along with a small quantity of *p*-cymene. In addition to linalool, the process can also be used for the deoxygenation/hydrogenation of terpene ethers including 1,8-cineole and 1,4-cineole. The mixture of *p*-menthane and 2,6-dimethyloctane has outstanding fuel properties including a higher gravimetric heat of combustion, lower viscosity, and acceptable density, compared to conventional jet fuel. These properties suggest that IHL has applications as a blending agent to enhance the properties of conventional jet fuels or existing sustainable aviation fuels.

To enable commercial production of sustainable aviation fuels from linalool, future research should focus on the synthesis of IHL at pilot-scale in parallel with work to improve the titer and conversion efficiency of linalool fermentation. Optimization of downstream processing and purification of linalool from the fermentation broth are other technical challenges that will need to be addressed. To produce the enormous volumes of jet fuel required for commercial air travel, a transition away from simple sugar feedstocks to lignocellulosic biomass will also be required. With regard to catalytic studies, it would be interesting to explore the use of Amberlyst-15/Pd catalysts for the dehydration/hydrogenation of additional monoterpene alcohols such as geraniol and nerol, as well as sesquiterpene alcohols including nerolidol. In addition, conducting the hydrogenation of the linalool dehydration mixture with non-noble metal catalysts may allow for increased sustainability of fuel production coupled with reductions in overall cost.

## Methods

### General

All glassware was dried in an oven at 140 °C prior to use. Amberlyst-15 H^+^ Form, 97% linalool, and 10% Pd/C were obtained from Sigma-Aldrich. All chemicals were used as received. ^1^H NMR spectra were collected as previously described^24^ and are displayed in Figures S4, S6, S8, S10, and S11. Experimental parameters for GC-FID, GC-MS, and the measurement of kinematic viscosity, density, and heat of combustion are included in the Supplementary Methods. The kinematic viscosity and density of IHL measured from 20 to −40 °C in 5 °C increments are listed in Table S1.

### Synthesis of IHL (2-pot procedure)

Amberlyst-15 H^+^ Form (43.70 g) was added to linalool (500 mL, 432.5 g, 2.804 mol) and the mixture was stirred in a 50 °C oil bath for one h. The composition of the C_10_ fraction as determined by GC-MS and GC-FID was 60% alkenes and 40% oxygenates. In addition, ~8% dimers were present in the product mixture. A portion of the dehydrated product (37.41 g) was transferred to a high-pressure reactor to which Amberlyst-15 H^+^ form (4.05 g) was added followed by 10% Pd/C (0.37 g). The reactor was attached to a hydrogen tank and vacuum line and subsequently evacuated and back-filled three times with hydrogen. Addition of the hydrogen resulted in an exotherm and an ice bath was used to keep the reactor temperature below 25 °C to prevent *p*-cymene formation. The pressure in the reactor was then increased to 500 psi H_2_ and the mixture was stirred at ambient temperature (20–25 °C) overnight. Next, the reaction was heated at 50 °C for 3 h, 100 °C for 3 h, and 150 °C for one h to facilitate dehydration/hydrogenation of etherous intermediates. The heat source was then removed and the reactor allowed to cool to ambient temperature. The contents of the reactor were filtered through Celite and the flask was rinsed with diethyl ether (2 × 50 mL). The filtrate was then transferred to a separatory funnel and the organic layer washed with a 10% w/v sodium bicarbonate solution (80 mL), distilled water (2 × 80 mL), and brine (1 × 80 mL). The aqueous layers were combined, filtered, and extracted with diethyl ether (1 × 50 mL). All of the organic extracts were combined, dried over MgSO_4_, and concentrated under reduced pressure. The crude product was then distilled via short path distillation under reduced pressure (bath temperature: 70 °C, 30 mm Hg, distillation head temperature: 28–30 °C) to yield 27.5 g (81%) of the product based on C_10_ components present in the dehydrated substrate. The product contained *p*-menthane (59.7%), 2,6-dimethyloctane (35.1%), and *p*-cymene (4.3%) as confirmed by GC-FID. A corresponding GC-MS trace can be found in Figure S5. Anal. Calc. for C_10_H_20.3_: C, 85.42; H, 14.58. Found: C, 85.04; H, 14.83.

### Synthesis of IHL (1-pot procedure)

Linalool (35.32 g, 0.229 mol), Amberlyst-15 H^+^ form (3.91 g), and 10% Pd/C (0.38 g) were added to a high-pressure reactor. The reactor was heated under air at 50 °C for 80 min. The heat source was then removed and the reactor was cooled to ambient temperature. The reactor was evacuated and back-filled as described above, pressured to 500 psi H_2_, and then stirred at 20–25 °C overnight followed by 50 °C for 3 h, 100 °C for 1 h, and 150 °C for 1 h. The reaction mixture was then worked up as described above to yield a crude product containing *p*-menthane and 2,6-dimethyloctane in roughly a 1:1 ratio, with 1% *p*-cymene and ~3% oligomerized products. The product was purified via short path distillation under reduced pressure (bath temperature: 70 °C, 30 mm Hg, head temperature: 28–30 °C) to yield 21.20 g (66% yield) of product. The distilled product contained *p*-menthane (51.9%), 2,6-dimethyloctane (43.5%), and *p*-cymene (1.2%), as confirmed by GC-FID. A corresponding GC-MS trace can be found in Figure S7.

### Deoxygenation/Hydrogenation of 1,8-Cineole

1,8-Cineole (23.31 g, 0.151 mol), Amberlyst-15 H^+^ form (2.38 g), and 10% Pd/C (0.26 g) were added to a high-pressure reactor. The reactor was connected to a hydrogen tank and vacuum line. The reactor was evacuated and back-filled as described above, pressured to 500 psi H_2_, and then heated to 50 °C until hydrogen uptake ceased, ~5 hours. Subsequently, the reaction temperature was increased to 100 °C and the reaction was left to stir overnight. The heat source was then removed and the reaction mixture was worked up as described above to yield a mixture of the product and diethyl ether. The solution was concentrated under reduced pressure to yield 29.18 g (84% yield) of product, which contained *p*-menthane (95.3%) and *p*-cymene (4.7%). A GC-MS trace of the product can be found in Figure S9.

### Deoxygenation/Hydrogenation of 1,4-Cineole

1,4-Cineole (44.00 g, 0.285 mol), Amberlyst-15 H^+^ form (4.51 g, 0.014 mol), and 10% Pd/C (0.44 g) were added to a high-pressure reactor. The reactor was connected to a hydrogen tank and vacuum line. The reactor was evacuated and back-filled as described above, pressured to 500 psi H_2_, and then stirred overnight at 50 °C. The temperature was then raised to 100 °C for 5 h. The heat source was removed and the mixture worked up as described above. The solution was concentrated under reduced pressure to yield 30.35 g (76% yield) of product. ^1^H NMR spectroscopy showed that the product was composed entirely of *p*-menthane.

## Supplementary information

Supporting Information

## Data Availability

Supplementary methods, ^1^H NMR spectra, gas chromatograms, a selected mass spectrum, and a kinematic viscosity data table can be found in the supporting information. Requests for additional data can be sent to the corresponding author.
