# Supplementary material for: Solvent-free dehydration, cyclization, and hydrogenation of linalool with a dual heterogeneous catalyst system to generate a high-performance sustainable aviation fuel
Source: Commun Chem. 2022 Sep 27;5:113. doi: 10.1038/s42004-022-00725-0 (PMC9814387; doi:10.1038/s42004-022-00725-0)
Supplement: Supplementary file 1 — Supporting Information [file 42004_2022_725_MOESM1_ESM.pdf]

Supporting Information for

Solvent-Free Dehydration, Cyclization, and Hydrogenation of Linalool with a Dual Heterogeneous Catalyst System to Generate a High-Performance Sustainable Aviation Fuel

C. Luke Keller, Karan R. Doppalapudi, Josanne-Dee Woodroffe, and Benjamin G. Harvey*

US NAVY, NAWCWD, Research Department, Chemistry Division, China Lake, California 93555

*Corresponding author (email: benjamin.g.harvey@navy.mil)

Contents

Supplementary Methods **S2**

NMR Parameters. **S2**

Fuel Characterization. **S2**

Kinematic Viscosity and Density Studies. **S2**

Heat of Combustion Studies. **S2**

Figure S1: Full GC-MS trace of isomerized linalool. **S3**

Figure S2: GC trace of isomerized linalool. **S4**

Figure S3. Mass spectrum of unknown oxygenated terpenoid eluting at ~8.5 min (GC-MS) **S5**

Figure S4: ^{1}H NMR spectrum of isomerized linalool. **S6**

Figure S5: GC trace of the final two-pot IHL fuel product. **S7**

Figure S6: ^{1}H NMR spectrum of the final two-pot IHL fuel product. **S8**

Figure S7: GC trace of one-pot IHL fuel product **S9**

Figure S8: ^{1}H NMR spectrum of the one-pot IHL fuel product. **S10**

Figure S9: GC trace of fuel product derived from 1,8-cineole **S11**

Figure S10: ^{1}H NMR spectrum of fuel product derived from 1,8-cineole. **S12**

Figure S11: ^{1}H NMR spectrum of fuel product derived from 1,4-cineole. **S13**

Table S1. Kinematic viscosity and density of isomerized hydrogenated linalool **S13**

Supplementary Methods

NMR Parameters. ^{1}H NMR spectra were recorded on a Jeol 400 MHz spectrometer at 25°C. Proton-decoupled ^{13}C{^{1}H} NMR spectra were recorded with the same instrument at 25 °C, operating at 100 MHz. Chemical shifts are reported in parts per million downfield from tetramethylsilane and are referenced relative to the NMR solvent ($CDCl_3$) according to the literature values–δ(^{1}H) = 7.26, δ(^{13}C) = 77.16.

Fuel Characterization. All GC-FID spectra were collected on an Agilent 7820A Gas Chromatograph (Santa Clara, CA). The GC oven method began at 40°C for 3 min, then ramped to 300°C at a rate of 10°C·min-1, and was held at 300°C for 1 min. The gas-phase separation was achieved using an Agilent Technologies DB-5 column, 60 m x .620 mm x 0.25 μm (Santa Clara, CA), the column was held at 12.59 PSI. A manual injection of 1 μL was injected for each run. The inlet's temperature was held at 250°C at a pressure of 12.59 PSI. Eluted compounds were fed into the Flame Ionization Detection (FID) which was held at the following parameters: a 300°C flame, a Hydrogen Gas flow at 40mL·min-1, an Air flow at 400mL·min-1, and a makeup gas flow at 27mL·min-1.

All GC-MS spectra were collected on a Thermo Scientific Trace 1310 Gas Chromatograph Exactive GC orbitrap mass spectrometer (San Jose, CA). The GC oven method began at 40°C for 3 min, then ramped to 300°C at a rate of 20°C·min-1, and was held at 300°C for 30 min. The gas-phase separation was achieved using a Thermo Scientific TG-5SILMS column, 30 m x .25 mm x 0.25 μm (San Jose, CA). A split flow mode of injection was used at 25 mL·min-1 with a split ratio of 25.0. The inlet temperature was held at 300°C. Eluted compounds passed through a heated auxiliary line (300°C) and were directed toward the ion source region of the mass spectrometer, held at 305°C, where they were subjected to electron impact ionization (EI). The mass range of the mass spectrometer was set from 50 to 600 amu.

Kinematic Viscosity and Density Studies. The kinematic viscosities and densities of the fuels were measured using a Stabinger Viscometer, SVM 3001, connected to a TC-502 chiller to achieve temperatures down to −40°C. Each sample was placed in a 5 mL syringe, which was then attached to the viscometer through a Luer Lock adapter. Approximately 3 mL of each fuel was then slowly injected to prewet the measurement cells. The sample was allowed to equilibrate at the starting temperature (typically 20°C). The method was then initiated, and an additional 1 mL of sample was added. Each sample was then cooled to −40°C (±0.002°C), and at 5°C increments, both the kinematic viscosity and density were measured. Reported values were derived from the average of five determinations. After each run, the measuring cells were rinsed three times with hexanes and dried under a stream of nitrogen.

Heat of Combustion Studies. The NHOCs of fuels derived from 97% Linalool were measured at NAWCWD using the following procedure. A pellet of high-purity benzoic acid (∼950−1000 mg) was accurately weighed, and ∼350−800 mg of fuel were added and allowed to fully saturate the pellet. The pellet was then reweighed, and the gross heat of combustion (HOC) was measured in a Parr 6200 Calorimeter. After combustion of the sample, the HOC was corrected by subtracting the contribution due to benzoic acid and combusted wire. The NHOC was then calculated from the corrected HOC by taking into account the hydrogen content (determined by elemental analysis, reported in **Table 1**) and the density of the fuel at 15°C. The NHOC measurements were taken in triplicate and averaged.

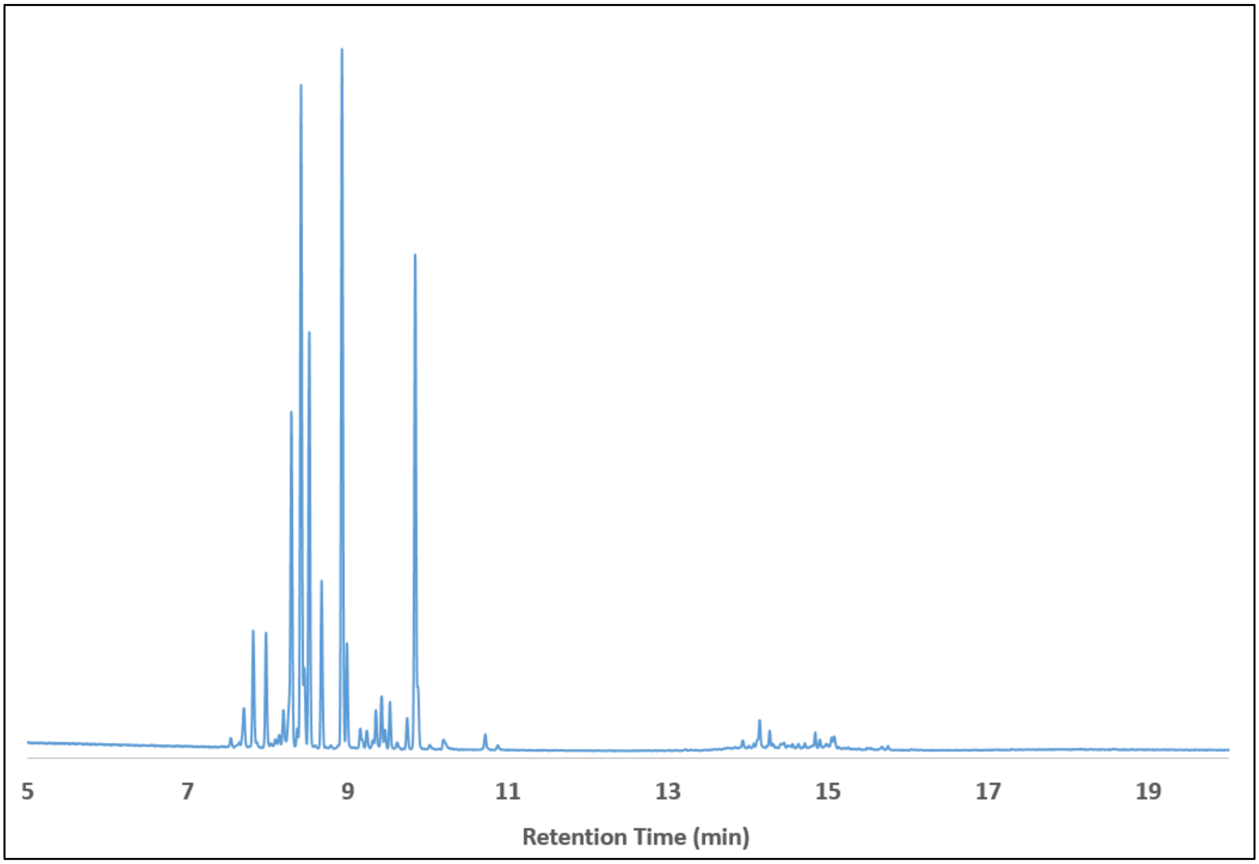

Figure S1. Full gas chromatogram (GC-MS) of dehydrated isomerized linalool. Peaks with retention times from ~7.5–11.0 min are C_{10} dehydration products, while peaks with retention times from ~13.5-16.0 min are C_{20} (dimer) products.

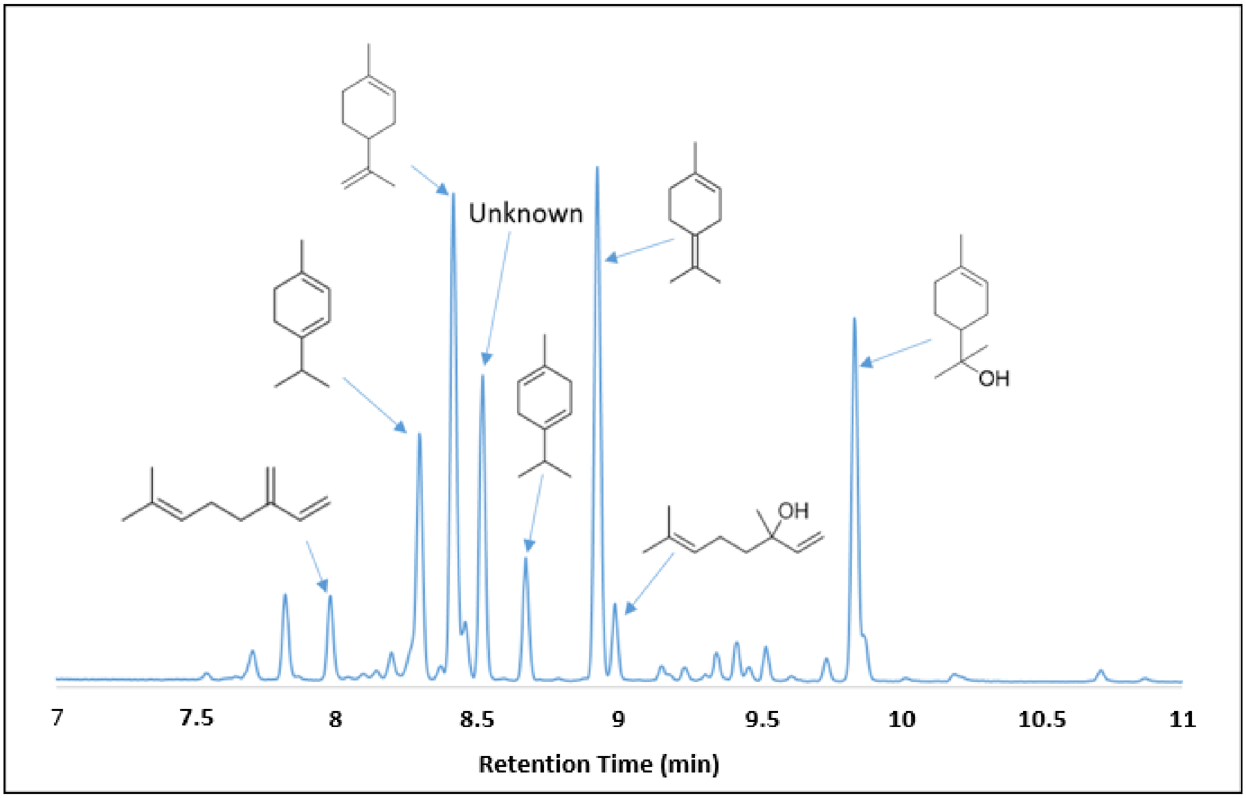

Figure S2. Gas chromatogram of isomerized linalool showing monoterpene products.

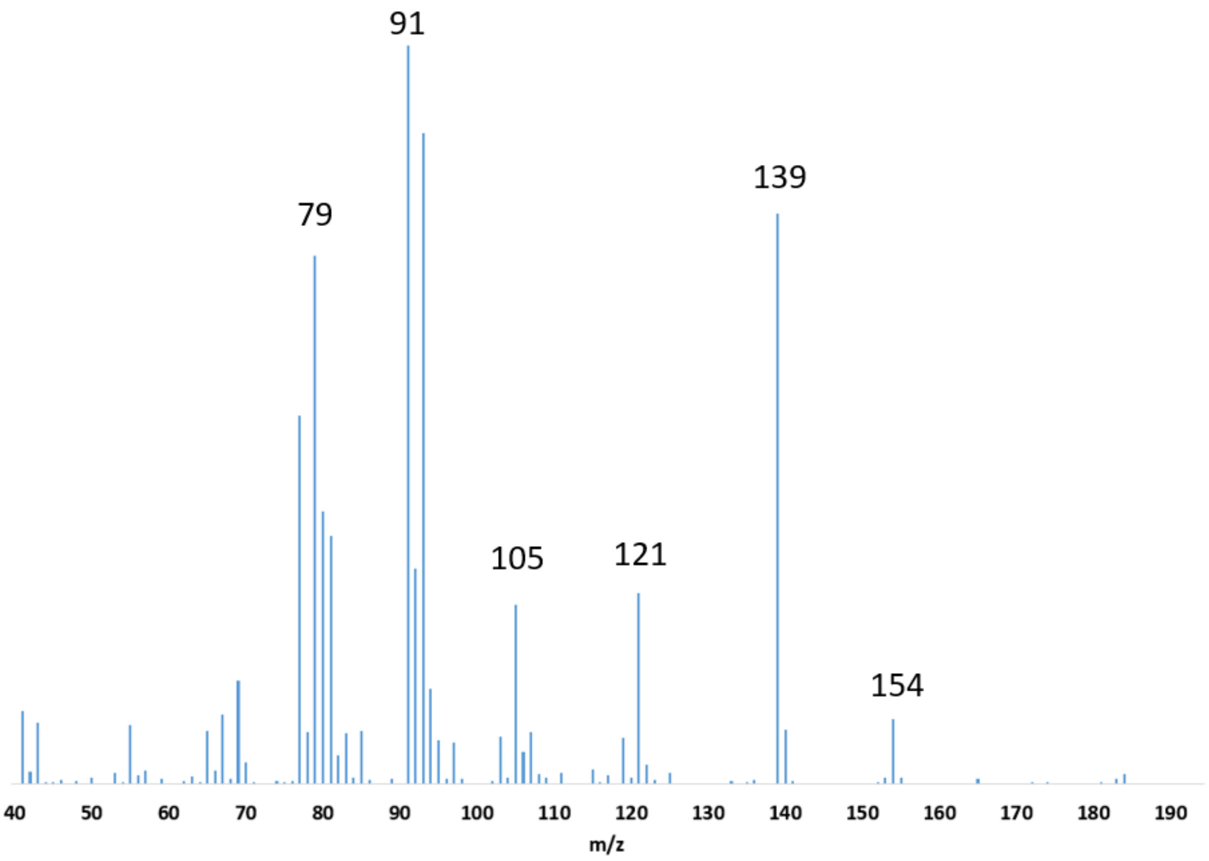

Figure S3. Mass spectrum of unknown oxygenated terpenoid eluting at ~8.5 min (GC-MS)

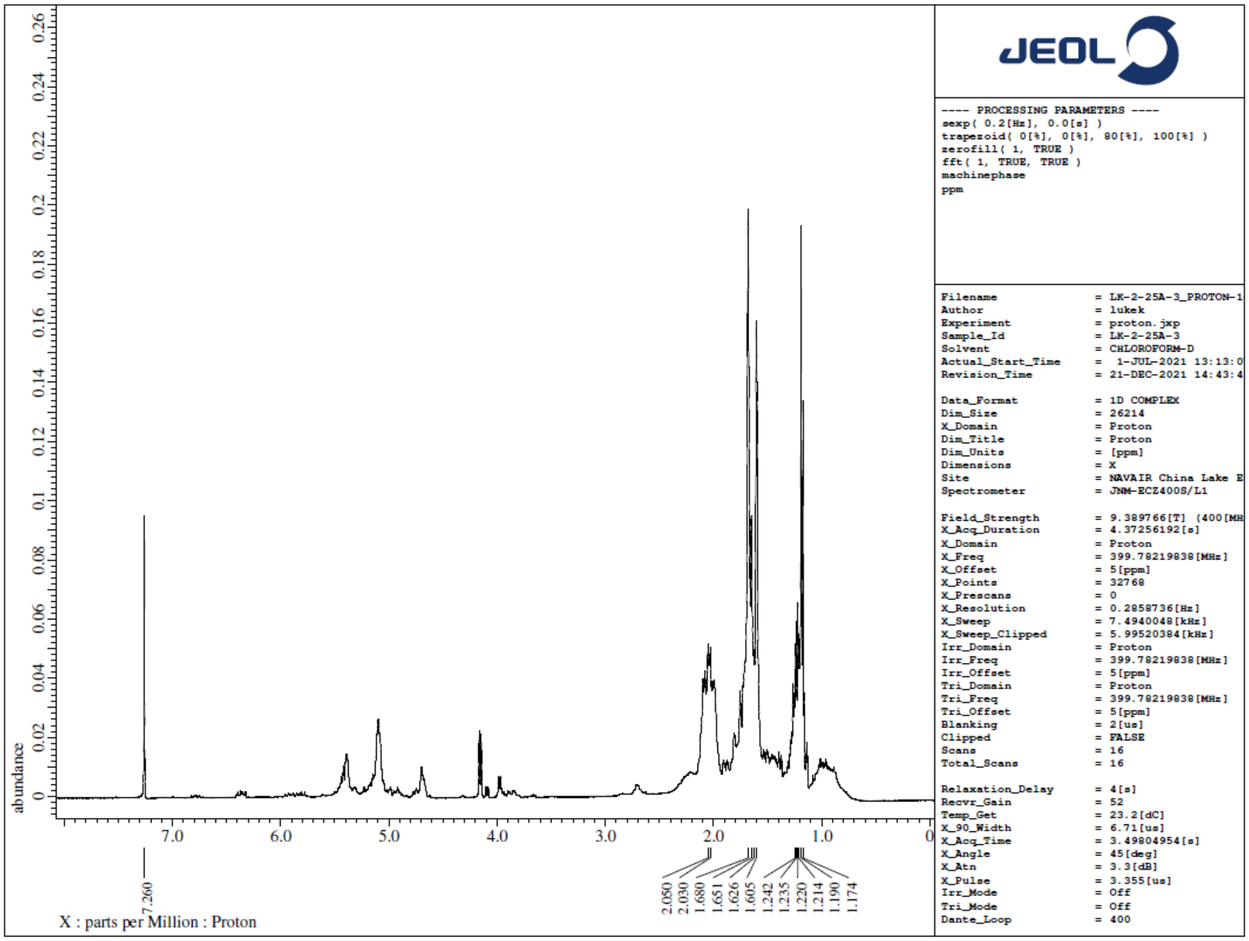

Figure S4, ^{1}H NMR spectrum (400 MHz, $CDCl_3$) of dehydrated isomerized linalool.

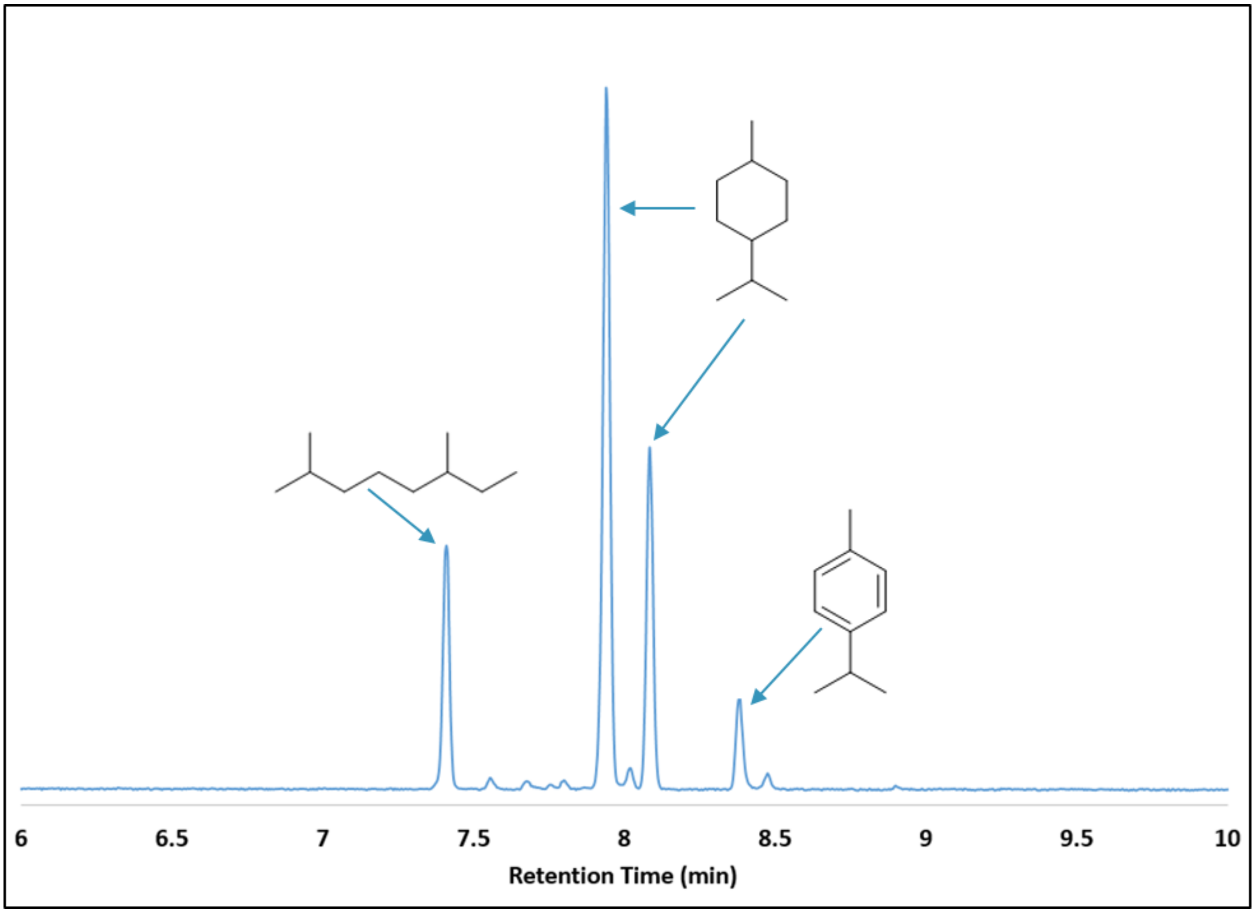

Figure S5. Gas chromatogram of isomerized hydrogenated linalool (two-step method).

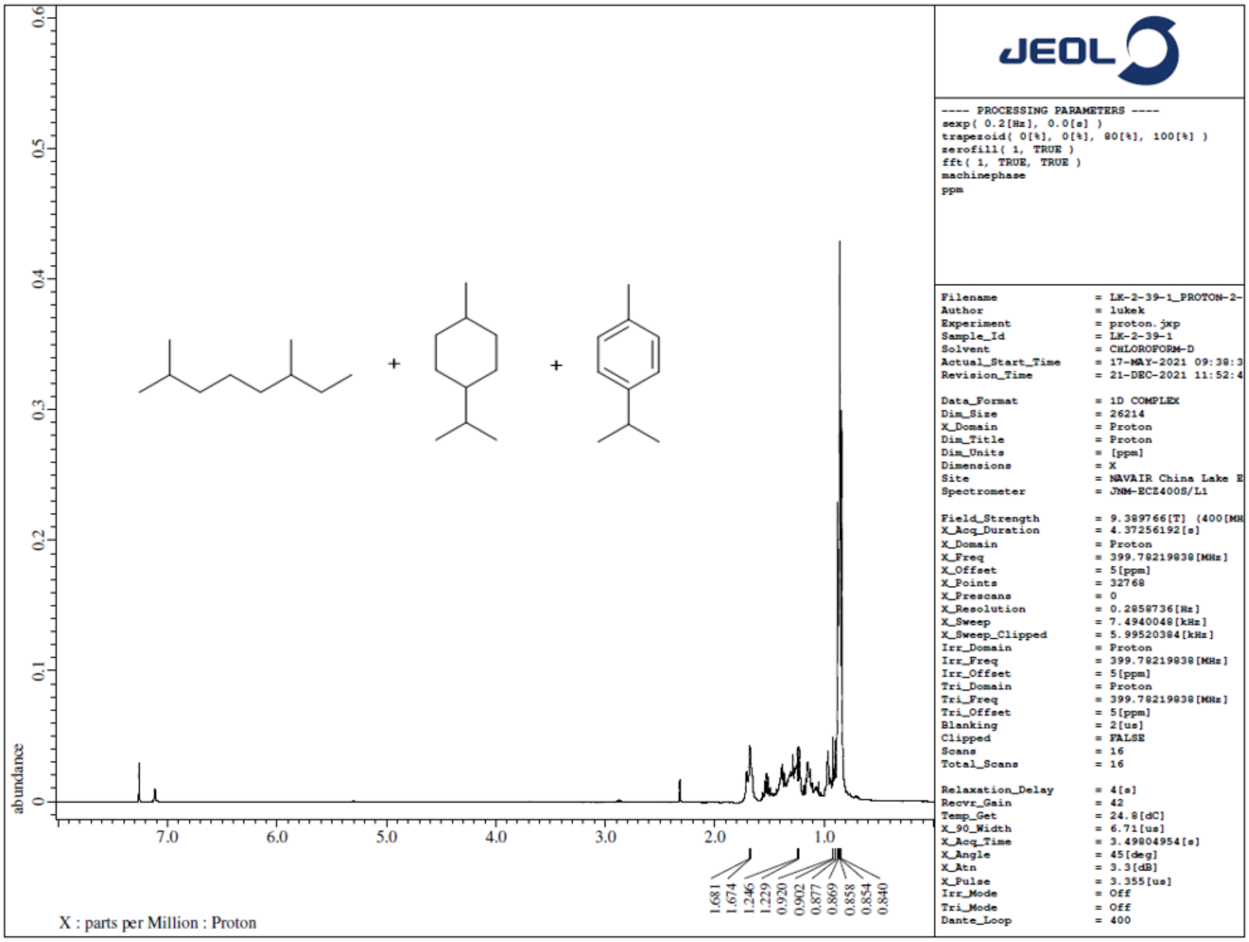

Figure S6. ^{1}H NMR spectrum (400 MHz, $CDCl_3$) of isomerized hydrogenated linalool (two pot method).

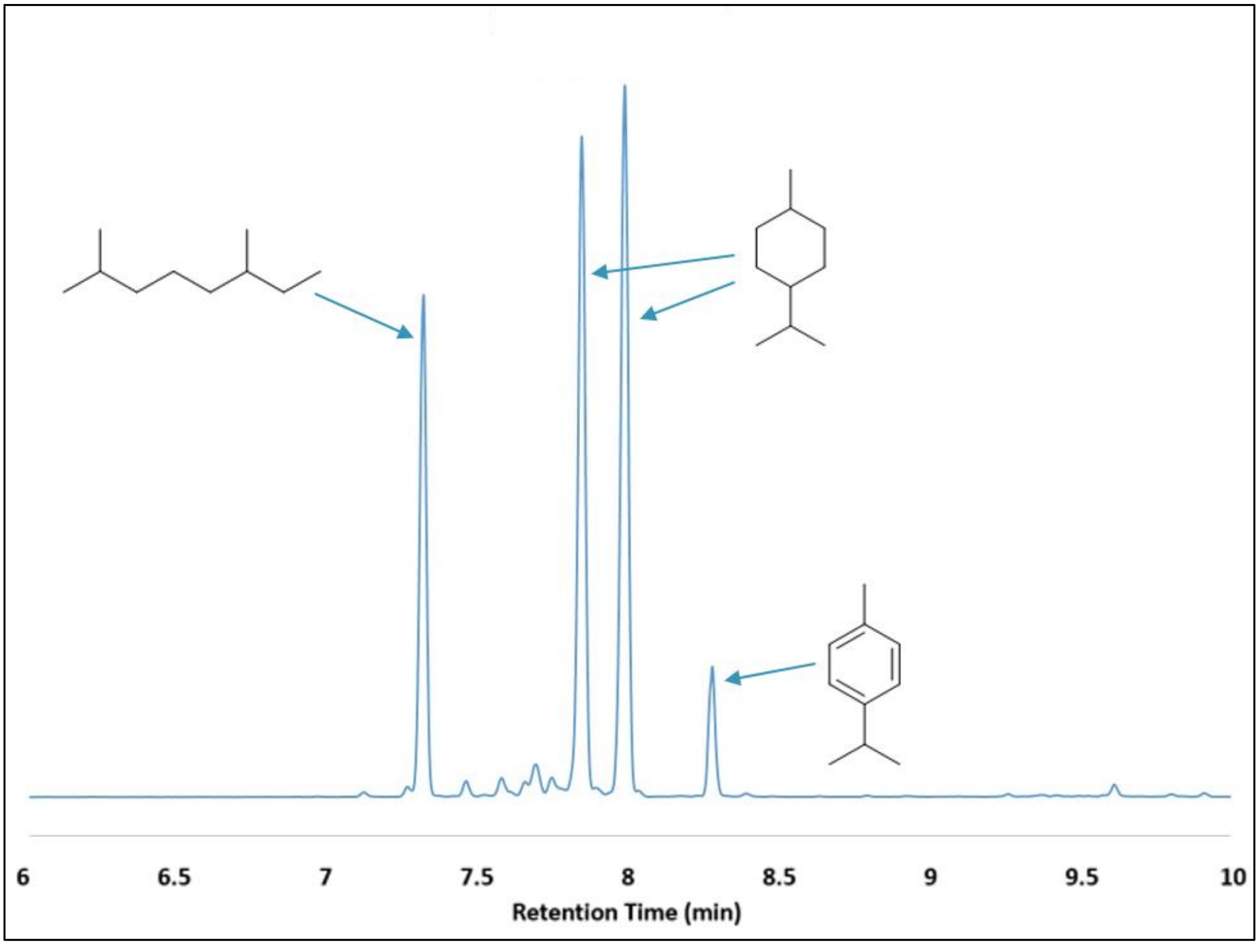

Figure S7. Gas chromatogram of isomerized hydrogenated linalool (one-pot method).

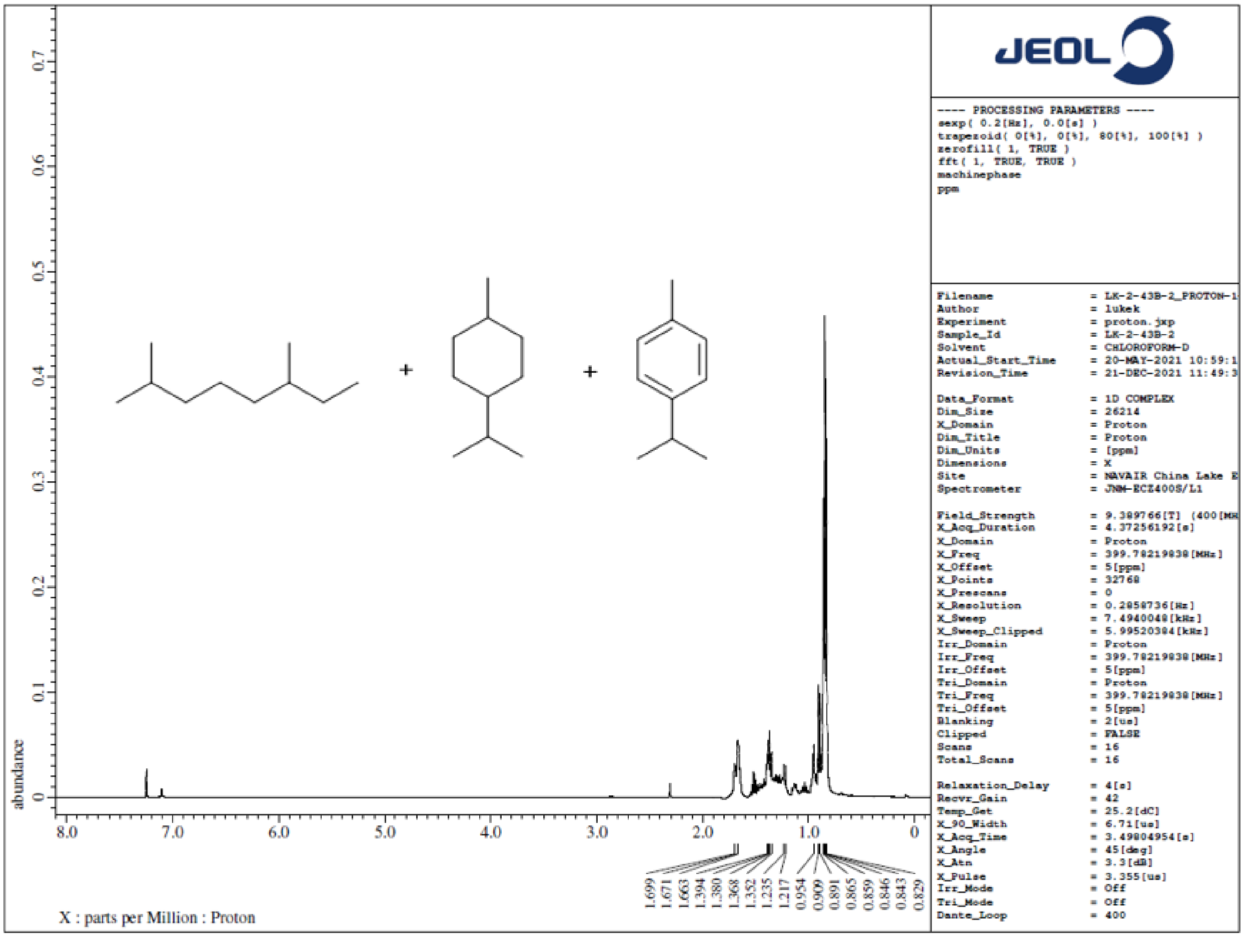

Figure S8: ^{1}H NMR spectrum (400 MHz, $CDCl_3$) of the one-pot IHL fuel product.

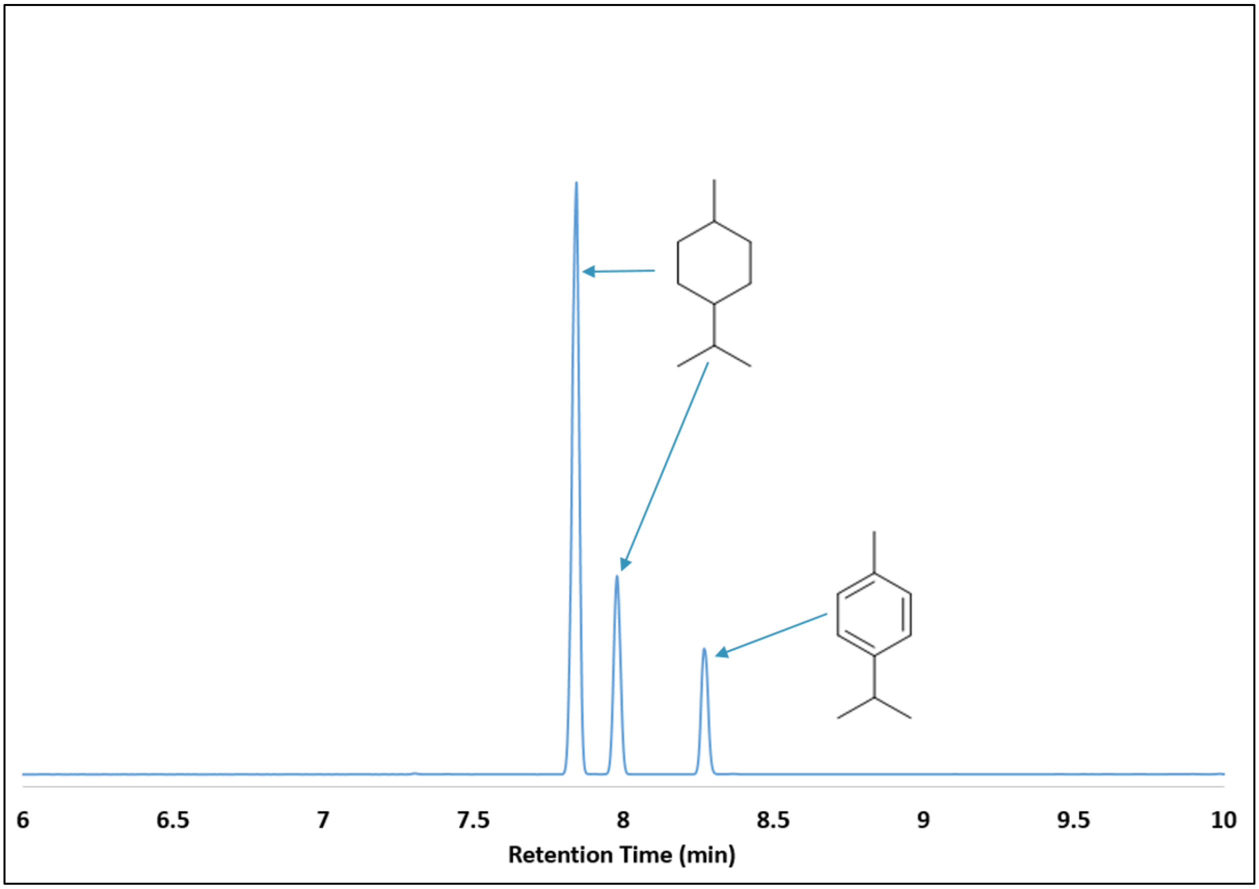

Figure S9: Gas chromatogram (GC-MS) of the product derived from deoxygenation of 1,8-cineole.

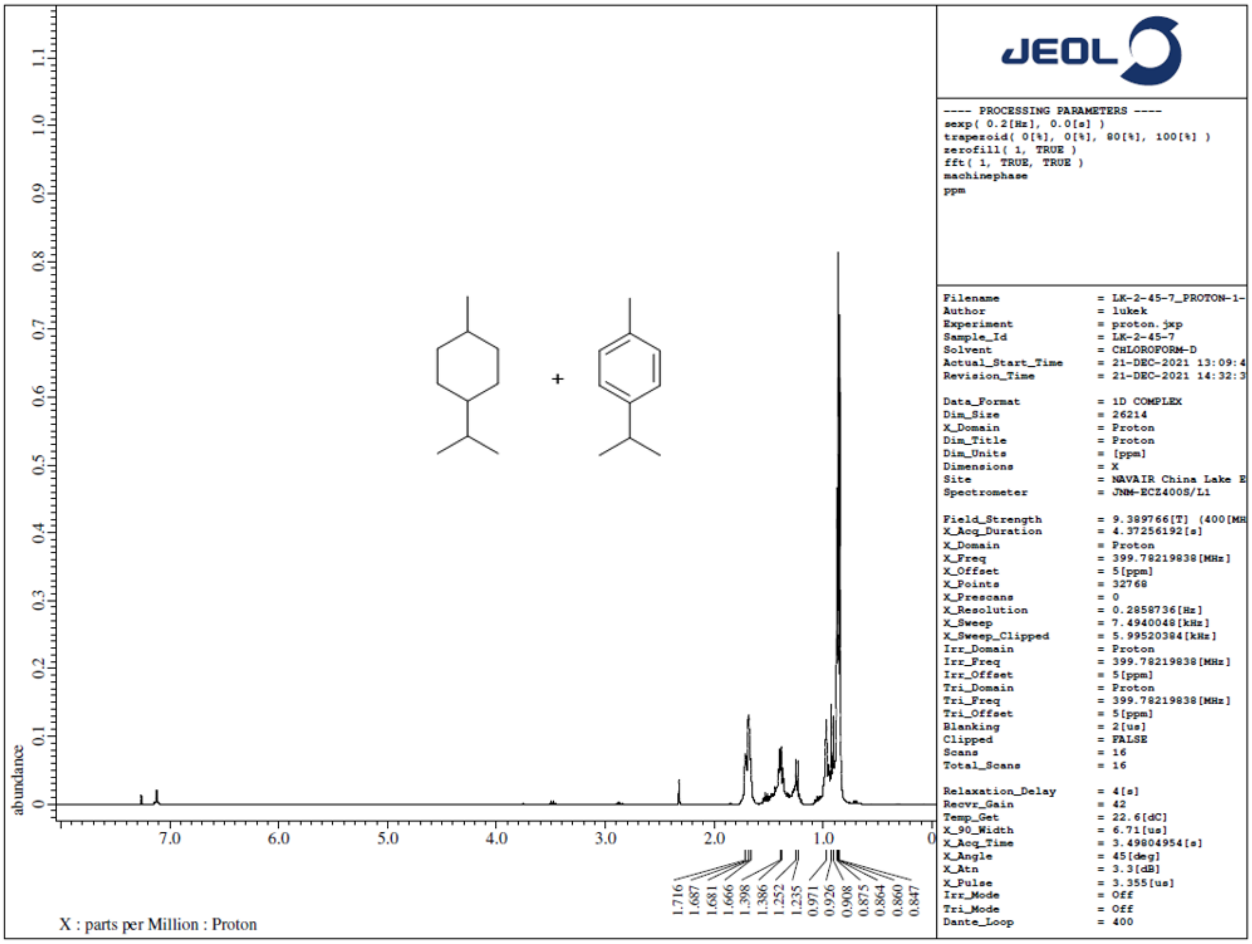

Figure S10. ^{1}H NMR spectrum (400 MHz, $CDCl_3$) of the product derived from deoxygenation of 1,8-cineole.

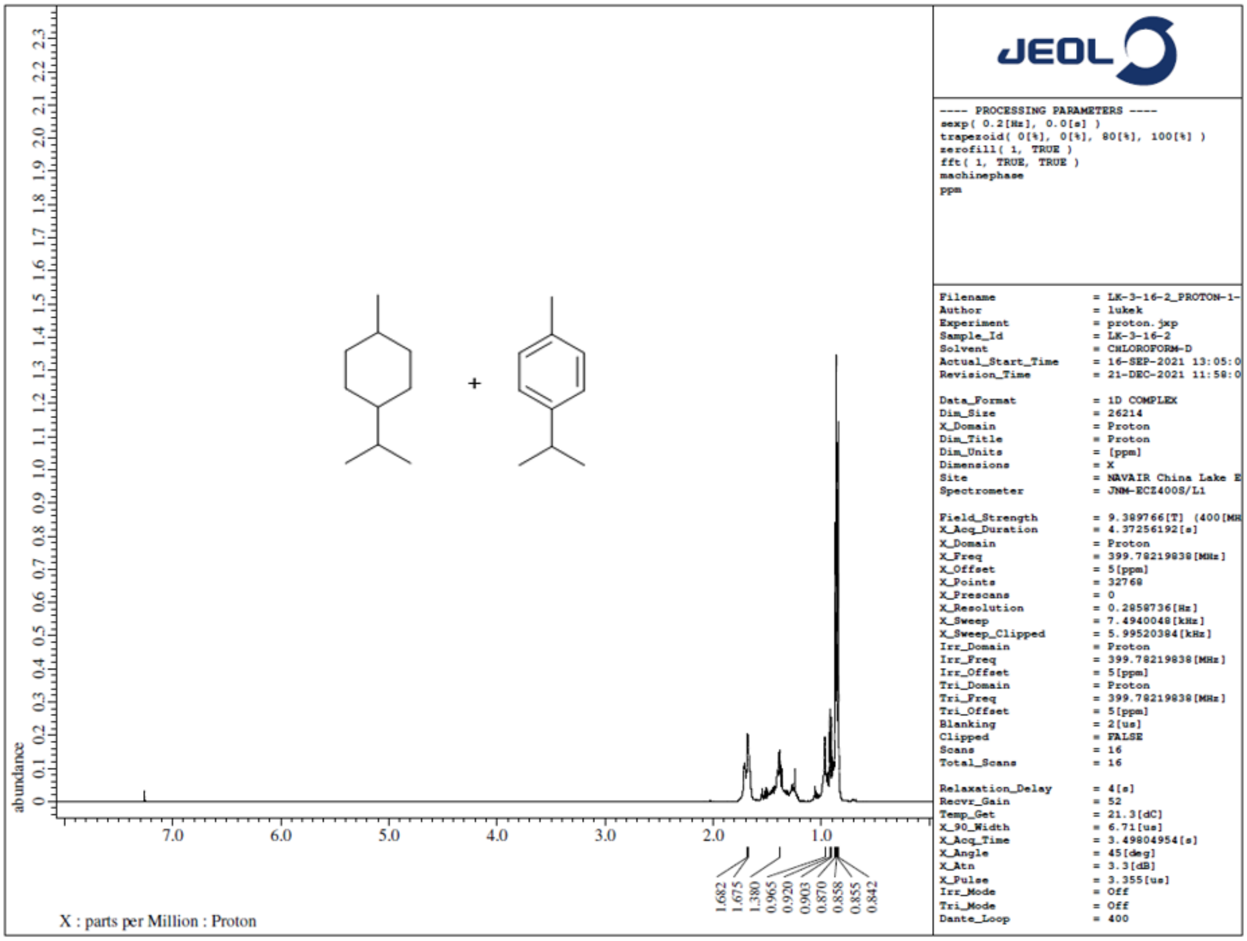

Figure S11. ^{1}H NMR spectrum (400 MHz, $CDCl_3$) of the product derived from deoxygenation of 1,4-cineole.

Table S1. Kinematic viscosity and density of isomerized hydrogenated linalool

Cell Temperature °C	Kinematic Viscosity (mm^2 s^{-1})	Density (g mL^{-1})
20.00	1.31	0.779
15.00	1.40	0.783
10.00	1.51	0.787
5.00	1.64	0.791
0.00	1.79	0.795
-5.00	2.00	0.798
-10.00	2.22	0.802
-15.00	2.47	0.806
-20.00	2.74	0.810
-25.00	3.13	0.813
-30.00	3.56	0.817
-35.00	4.09	0.821
-40.00	4.74	0.824
